# Development and psychometric properties of a questionnaire to evaluate the professional behavior of medical residents

**DOI:** 10.1371/journal.pone.0358787

**Published:** 2026-09-28

**Authors:** Shahsanam Gheibi, Mohammad Heidari, Alireza Alemi, Parsa Shadnezhad, Rahim Mahmodlu, Ali Parvin, Seyed Fahim Irandoost

**Affiliations:** 1 Maternal and Childhood Obesity Research Center, Urmia University of Medical Sciences, Urmia, Iran; 2 Department of Epidemiology and Biostatistics, School of Medicine, Urmia University of Medical Sciences, Urmia, Iran; 3 Student Research Committee, Urmia University of Medical Sciences, Urmia, Iran; 4 Department of General Surgery, Urmia University of Medical Sciences, Urmia, Iran; 5 Education Development Center, Urmia University of Medical Sciences, Urmia, Iran; 6 Department of Community Medicine, School of Medicine, Urmia University of Medical Sciences, Urmia, Iran; Khoy University of Medical Science: Khoy University of Medical Sciences, IRAN, ISLAMIC REPUBLIC OF

## Abstract

Given the critical ways in which medical residents’ professional behavior affects public health and the quality of care, this study aimed to develop and evaluate the psychometric properties of a questionnaire assessing the professional behavior of medical residents in Iran. Initially, an item pool was created based on a literature review and analysis of relevant questionnaires, particularly the Guidelines and Evaluation Tools for Adherence to Professional Principles by Medical Residents. The instrument’s psychometric properties were then assessed through content validity, face validity, construct validity, and reliability. Data were analyzed using SPSS-22 and STATA-16. The initial version included 62 items. Content validity, evaluated by 25 experts across health and medical fields, yielded a Content Validity Ratio (CVR) >0.37 and a Content Validity Index (CVI) >0.79, leading to the removal of 13 items. Face validity, assessed with 12 medical residents via the impact score method, led to the elimination of 3 additional items. Construct validity, examined with 217 medical residents, identified six factors explaining approximately 84.7% of the total variance. Exploratory and confirmatory factor analyses supported a six-factor structure of the final 18-item instrumen. Reliability was confirmed through internal consistency (Cronbach’s alpha = 0.94) and stability (Intraclass Correlation Coefficient = 0.83). The final questionnaire provides a valid and reliable tool for evaluating the professional behavior of medical residents and general medical students in clinical settings. It is recommended that educators, administrators, and healthcare policymakers utilize this instrument to assess professional conduct, identify areas for improvement, and design targeted interventions to enhance professional behavior in medical training.

## Introduction

Professionalism is vital for physicians, as it impacts healthcare quality and prestige [1,2]. A lack of professionalism can lead to numerous adverse outcomes, such as lower healthcare quality, heightened dissatisfaction, conflicts, and violence, while diminishing the value and prestige of the medical profession. This particularly undermines the public’s trust in the healthcare system [3,4]. Consequently, assessing professionalism is crucial in medical education [5,6]. One key responsibility of medical schools is to assess whether these competencies have been met [7]. Additionally, it is essential to train medical residents to interact professionally with patients [8–14].

Professionalism lacks a unified definition or universally accepted framework, as different perspectives have led to diverse conceptualizations and approaches to teaching and assessment [12,13]. Recognizing this diversity, the International Working Group on the Assessment of Professionalism emphasized the multidimensional nature of the construct and recommended that evaluations encompass individual, interpersonal, and social institutional domains [15]. Since no single method can fully capture all aspects of professionalism, scholars advocate using multiple complementary approaches to ensure a comprehensive assessment [16]. Over the past three decades, numerous tools have been developed to assess professionalism in medical trainees [17–19], yet concerns remain regarding the lack of universally valid and reliable measurement standards. Moreover, because the behavioral expression of professionalism varies across sociocultural contexts, instruments developed in one setting may not be fully applicable in another.

Several influential frameworks have shaped the theoretical understanding of medical professionalism. Cruess and Cruess conceptualize professionalism as a sociocultural contract between medicine and society, grounded in integrity, accountability, and a commitment to competence [20, 21]. The ABIM Physician Charter similarly highlights principles such as respect, altruism, and prioritizing patient welfare [22]. Hodges et al. (2011) describe professionalism as a multilevel construct that includes personal attributes, interpersonal behaviors, and system-level responsibilities [15], while Van De Camp et al. (2004) underscore the influence of individual, relational, and societal expectations [12]. In parallel, the quality of professionalism assessment tools is often judged based on their reliability, validity, feasibility, acceptability, and educational impact [23–25]. Among the existing methods, direct observation in authentic clinical settings has been considered particularly effective, leading to the widespread use of instruments such as the Professionalism Mini-Evaluation Exercise (P-MEX), which evaluates doctor–patient relationships, reflective skills, time management, and interprofessional interactions [20,26]. Despite these advancements, no universally accepted standard exists for systematically evaluating the measurement quality of professionalism instruments, limiting confidence in their applicability across contexts [19]. To achieve a focused and measurable construct definition, our instrument is conceptually organized around six dimensions derived from integrating major international frameworks with the specific context of the Iranian National Professionalism Guidelines. This integration ensures that the tool captures both universal ethical principles and local behavioral expectations. These six dimensions, responsibility and accountability, adherence to regulations, respect for others, integrity and honesty, professional excellence, and altruism, collectively represent the core domains of professional behavior, encompassing individual, interpersonal, and systemic responsibilities as highlighted by hodges et al. specifically, they cover the commitment to competence, accountability, and the prioritization of patient welfare, thereby providing a clear operational definition for the assessment [27,28]

Although several frameworks and tools have been developed internationally, most existing professionalism instruments rely on evaluations by faculty, peers, or patients rather than assessing residents’ own perceptions of their professional behavior. Widely used tools such as P-MEX and multisource feedback systems primarily measure externally observable behaviors, and recent reviews highlight the lack of validated self-assessment instruments targeted specifically at residents [19,26]. This gap is particularly relevant in cultures where norms, expectations, and expressions of professionalism differ, as residents may internalize and interpret professional responsibilities in ways not fully captured through external evaluation [29]. In Iran, despite the use of several professionalism-related questionnaires, none have been psychometrically validated to measure residents’ professionalism from their own perspective.

Although numerous instruments have been developed to assess physicians’ professional behavior, most rely on evaluations from patients, nurses, colleagues, or faculty members rather than capturing residents’ own perceptions or the complex challenges they navigate during training [30–32]. This gap is particularly important in Iran, where the cultural and organizational context of medical education shapes the manifestation of professionalism in distinct ways. The hierarchical structure of teaching hospitals, strong emphasis on adherence to institutional regulations, and sociocultural expectations regarding respect, modesty, and communal responsibility influence how professional behavior is interpreted and enacted by residents. Despite these contextual features, no resident-centered, culturally grounded, psychometrically validated instrument currently exists in Iran. Guided by multidimensional professionalism frameworks [15,28] and the national professionalism guidelines of the Iranian Ministry of Health [27], we conceptually anticipated that residents’ professional behavior would be organized around domains such as Responsibility, Adherence to Regulations, Respect, Integrity, Excellence, and Altruism. However, existing instruments may not necessarily capture all aspects of professional behavior that are relevant to the educational, cultural, and clinical context of Iranian medical residents. This highlights the need for a contextually appropriate instrument specifically developed to assess professional behavior in this population. Accordingly, this study aimed to develop and validate a theoretically informed and culturally sensitive instrument to enhance the assessment of professionalism within the Iranian medical education context.

## Methods

The present study is part of a broader research project entitled *Investigating the Relationship between Job Satisfaction and Professional Behavior among Medical Residents: A cross-sectional study in Urmia, Iran*, which received ethical approval from the Ethics Committee of Urmia University of Medical Sciences. All procedures involving human participants were performed in accordance with the ethical standards of the institutional and/or national research committee and with the 1964 Helsinki Declaration and its later amendments or comparable ethical standards. To minimize the risk of harm to participants, the study was designed as an anonymous, voluntary questionnaire-based investigation with no intervention or manipulation of clinical or educational activities. Participants were informed that their responses would remain confidential, would be analyzed in aggregate form only, and would not influence their academic evaluation, residency standing, or professional relationships. No identifiable personal information was collected. The potential risks associated with participation were considered minimal and primarily limited to the time required to complete the questionnaire and possible mild discomfort in reflecting on professional behaviors. The anticipated benefits of the study, including the development of a culturally grounded and psychometrically valid instrument for assessing professionalism among medical residents, were considered to outweigh these minimal risks. The resulting instrument may contribute to improving professionalism assessment, educational planning, and the quality of medical training and patient care. Written informed consent was obtained from all participants before enrollment. Participants received information regarding the study objectives, procedures, voluntary nature of participation, confidentiality measures, and their right to withdraw from the study at any time without any consequences. This methodological study was conducted to develop and psychometrically evaluate an instrument. Data collection, including the literature review, development of the questionnaire, and administration of the questionnaire to participants for completion, was carried out between 25 September 2023 and 29 February 2024.

### Phase One: Designing the Initial Version of the Instrument

Several methods were employed to design the initial version of the instrument (item bank). These included a literature review, examination of similar and related questionnaires that assess professional behavior from the perspectives of patients, colleagues, and faculty members, with an emphasis on the checklist of the ‘Guidelines and Evaluation Tools for Assessing the Professionalism of Medical Residents’ issued by the Ministry of Health, Treatment, and Medical Education of Iran [27]. The Ministry’s checklist focuses on assessing the professional behavior of medical residents from the viewpoint of faculty members. The item bank was developed based on these sources, particularly the Guidelines and Evaluation Tools for Assessing the Professionalism of Medical Residents,’ and was formulated in a declarative format.

During item generation, we adopted both procedural and conceptual decision-making approaches to ensure that the emerging item pool reflected a coherent theoretical and cultural foundation. Guided by national guidelines on professional conduct and established professionalism frameworks (Cruess & Cruess; the ABIM Physician Charter; Hodges’ multilevel model), we formulated an implicit theoretical proposition that residents’ professional behavior in the Iranian context constitutes a multidimensional construct aligned with six conceptually defined domains: conscientiousness and responsibility, adherence to the laws, respect for others, integrity and honesty, professional excellence, and altruism. Beyond quantitative criteria such as CVR, CVI, and impact scores, cultural considerations specific to Iranian residency training, including the hierarchical structure of teaching hospitals, strong expectations for compliance with institutional regulations, and sociocultural norms emphasizing respectful communication, modesty, and communal responsibility, were explicitly incorporated into the item selection process. Each candidate’s behavior was retained only if it mapped clearly onto one or more theoretically grounded domains and represented a meaningful and culturally relevant expression of professionalism within Iranian clinical training. This combined theoretical and culturally informed framework ensured that item development extended beyond mechanical validation procedures and remained anchored in conceptual coherence.

### Phase Two: Psychometric evaluation of the instrument

This phase was designed and implemented in four stages.

### Content Validity

Content validity was assessed both qualitatively and quantitatively to evaluate the components of the questionnaire. In the qualitative aspect, the questionnaire was provided to 25 experts who, after a thorough review, were asked to provide detailed and written feedback on the observance of grammar, use of appropriate words, placement of items in their proper positions, and suitable scoring.

The quantitative evaluation of content validity was conducted using the content validity ratio (CVR) and content validity index (CVI) [33]. To calculate the CVR, experts were asked to rate each item on a three-point scale: essential, valuable but not essential, and not essential. The numerical value of CVR ranges from +1 to −1, and since the number of experts was 25, based on the “minimum value determination” table by Lawshe, the minimum acceptable CVR score for an item considered essential was 0.37.

$\text{CVR}=\frac{n_{E}-\frac{N}{2}}{\frac{N}{2}}$

To calculate the CVI, three criteria—simplicity, relevance, and clarity—were individually evaluated for each item using a 4-point Likert scale (1 = not simple to 4 = completely simple) by the experts. The CVI was calculated by considering the total number of agreements for each item that received a score of 3 or 4 (the highest score). Items with a CVI score above 0.79 were deemed appropriate, those between 0.79 and 0.70 required revision, and items with a score below 0.70 were considered unacceptable and were removed [34].

$CVI\;=\;\frac{The\;ratio\;of\;the\;number\;of\;evaluators\;who\;gave\;the\;scores\;of\;\text{3}\;and\;\text{4}\;to\;an\;item}{Total\;number\;of\;evaluators}$

### Face Validity

Face validity was assessed both qualitatively and quantitatively [35]. For the qualitative assessment, the questionnaire was read to 12 individuals from the target population one by one, and their understanding of each item was inquired about. This process examined the difficulty in understanding the items and words, the relevance of the items to the dimensions of the questionnaire, and the presence of any ambiguity (i.e., the possibility of misinterpretation or inadequate meaning of words). Necessary revisions were made as needed.

For the quantitative assessment of face validity, the “impact score” method was used to reduce and eliminate inappropriate items and to determine the importance of each item. A 5-point Likert scale (5 = very important to 1 = not important at all) was used for each item. Twelve participants were asked to review and choose one option for each item. Items with an impact score greater than 1.5 were retained, while the others were eliminated [36].


$$\begin{array}{c} \text{Importance}\times \text{Frequency (as a percentage) = Impact Score} \end{array}$$

### Construct Validity

Although some authors suggest that a sample size between 100 and 300 participants is generally adequate for factor analysis regardless of the number of items [36], a census sampling strategy was used in the present study. The target population for the construct validity phase comprised all medical residents enrolled at Urmia University of Medical Sciences during the study period. Accordingly, all 256 eligible residents across the university’s residency programs were invited to participate. Participation was voluntary, and residents were informed about the study objectives and their rights as participants before completing the questionnaire. Of the 256 eligible residents, 217 completed the questionnaire, yielding a response rate of 84.8% (Table 3). Because all eligible residents at the institution were invited, the sample provided comprehensive coverage of the resident population at Urmia University of Medical Sciences. Nevertheless, the single-institution setting may limit the generalizability of the findings to medical residents in other universities and regions of Iran. Sampling adequacy and the suitability of the correlation matrix for factor analysis were confirmed through the Kaiser–Meyer–Olkin (KMO) measure and Bartlett’s Test of Sphericity.

Construct validity was reassessed using Exploratory Factor Analysis (EFA) with Principal Component Factoring. Factor retention was conceptually guided by the a priori six-domain theoretical framework and confirmed by the Scree plot inflection point, while items demonstrating standardized factor loadings > 0.40 were retained. To provide rigorous evidence of construct validity, a Confirmatory Factor Analysis (CFA) was conducted to test the fit of the 18-item, six-factor model. Model fit was evaluated using the Chi-square ratio (\chi^2 {df}), Comparative Fit Index (CFI), Tucker-Lewis Index (TLI), Root Mean Square Error of Approximation (RMSEA), and Standardized Root Mean Square Residual (SRMR).

### Reliability

Test-retest reliability was used to assess the questionnaire items. The questionnaire was administered twice with a 10-day interval to 20 medical residents, and the intraclass correlation coefficient (ICC) was calculated. Additionally, to assess internal consistency, the questionnaire was completed by 217 participants from the target group, and Cronbach’s alpha was calculated. A minimum Cronbach’s alpha of 0.7 was considered acceptable [37]. The 10-day interval for test–retest reliability was selected to minimize recall bias while ensuring that residents’ attitudes and perceptions would not meaningfully change over a short period. This time frame aligns with recommendations in psychometric methodology for attitudinal constructs. The choice of ICC (two-way mixed, absolute agreement) was conceptually driven by the need to assess the stability of individual scores rather than the consistency of raters. Data were collected and analyzed using SPSS software (Version 22, SPSS Inc., Chicago, IL, USA) and STATA-16.

Overall, although psychometric indices played an important role in guiding the refinement process, the instrument’s development was ultimately grounded in conceptual reasoning. Decisions at each step, including item retention, modification, factor interpretation, and reliability evaluation, were made through an integrated approach that combined theoretical relevance, qualitative insights, and psychometric evidence. This ensured that the final scale reflected both methodological rigor and conceptual integrity.

### Scoring and Interpretation

The total score and domain-specific scores are calculated as the mean of the respective items, ranging from 1 to 5. To facilitate interpretation in clinical settings, scores were classified into three levels of professional adherence based on responses recorded using a 5-point Likert scale ranging from 1 (strongly disagree) to 5 (strongly agree).

## Results

Based on the methods, the results are presented in two main phases.

### Phase One: Designing the Initial Version of the Instrument

In this phase, the item bank for the Professional Behavior Questionnaire was developed with 62 items, categorized into six dimensions: conscientiousness and responsibility, adherence to the laws, respect for others, integrity and honesty, professional excellence, and altruism.

### Phase Two: Psychometric Evaluation of the Instrument

The results of the psychometric evaluation are as follows:

### Content Validity

To assess qualitative content validity, 25 experts (physicians from internal medicine, pediatrics, neurology, obstetrics and gynecology, and other fields, as well as faculty members familiar with tool development in medical informatics, social medicine, medical education, health education, social welfare, etc.) assessed the quality of the items. Based on their feedback, necessary revisions were made to the items. In the quantitative evaluation of content validity, based on the CVR, 12 items (5, 13, 27, 28, 29, 30, 32, 43, 55, 59, 61, and 62) received a score below 0.37 and were removed. For the CVI, eight items scored below 0.70 and were also removed; seven overlapped with items already removed based on the CVR, and the newly eliminated item was item 2. According to the CVI results, items 10, 27, 28, 43, 44, 52, and 60 required revisions (Table 1), and these items were subsequently reviewed and revised for wording and phrasing (Table 2). Additionally, some items were reclassified under different dimensions based on expert opinion. Moreover, the experts proposed a few new items, and some items were split into several distinct items (Table 2).

**Table 1:** Results of Impact Score, Content Validity Ratio (CVR), and Content Validity Index (CVI)*.

No	Items	Impact score	CVR	CVI
1	I ensure that patient care is followed up at different stages.	3.4	1	0.89
2	With an awareness of the limitations of each diagnostic method and the success rate of each treatment approach, I provide the best possible care for the patient.	3.4	0.76	0.66
3	I perform assigned duties correctly.	2.91	0.76	0.90
4	I am accessible to patients and colleagues.	2.22	0.44	0.88
5	If needed, I assist my colleagues.	1.88	0.28	0.90
6	I use the resources and facilities of the healthcare system appropriately.	3.24	0.52	0.8
7	I am committed to educating junior residents and other learners.	4.16	0.84	0.94
8	I accurately document patient status or care provided (hospital, clinic, or educational documentation).	3.07	0.84	0.94
9	I make decisions about patient treatment based on my professional knowledge, skills, and experiences.	3.56	0.44	0.82
10	I carry out the care that I have planned for the patient.	3.07	0.44	0.74
11	I provide the patient or, if necessary, their family with the information about the illness, understandably and patiently.	4.09	0.84	0.94
12	I expedite the provision of medical care for the patient under my supervision or the delivery of medical consultations.	2.62	0.52	0.82
13	I prioritize multiple roles and responsibilities to succeed in all areas.	3.07	0.2	0.58
14	I submit assignments and practical tasks on time.	0.4	0.52	0.82
15	I arrive on time at the ward.	2.9	1	1
16	I arrive on time for meetings, classes, and field activities.	2.44	1	0.96
17	I adhere to the attendance and absenteeism policies.	2.9	0.44	0.94
18	If I need to be absent or delayed, I inform professors, supervisors, colleagues, or patients in a timely and appropriate manner.	3.3	0.84	0.96
19	I maintain a dress code and appearance appropriate for the clinical and care environment.	3.91	0.84	0.92
20	I adhere to hospital rules and regulations.	3.56	1	0.97
21	I report illegal actions.	0.79	0.44	0.94
22	I report unethical actions.	1.3	0.76	0.94
23	I treat the patient with respect.	3.54	0.92	1
24	I provide services with respect.	2.37	0.92	0.94
25	I respect my colleagues.	1.3	0.76	0.94
26	I do not use derogatory language when interacting with others.	3.15	0.84	0.94
27	I strive to present information concisely and effectively, avoiding unnecessary verbosity.	3.91	0.12	0.74
28	I provide constructive feedback to my colleagues.	4.5	0.28	0.78
29	I demonstrate a positive attitude towards others through my body language.	4.16	0.12	0.54
30	I make decisions based on collecting sufficient and accurate data about the facts.	4.09	0.28	0.56
31	I listen attentively to the patient.	2.64	0.6	0.85
32	I use appropriate verbal and non-verbal body language based on the context.	3.07	0.04	0.54
33	I maintain eye contact with the patient.	2.36	0.52	0.82
34	I treat the patient as a person (not an object or case).	3.5	0.92	0.92
35	I maintain my composure in difficult situations.	3.38	0.68	0.92
36	Patient safety is a priority for me.	2.4	0.84	0.90
37	I maintain patient confidentiality	1.7	1	1
38	I respect patients’ privacy.	3.45	1	1
39	I protect the confidentiality of patient information.	2.29	0.84	0.88
40	I respect the patient’s right to refuse treatment and care.	3.8	0.68	0.85
41	I am honest in my interactions with my colleagues.	4.91	0.76	0.89
42	I am considered a reliable person by my colleagues and patients.	4.01	0.52	0.93
43	I focus on patients’ needs.	4.41	0.28	0.74
44	I pay attention to the patient’s needs during the examination.	2.74	0.44	0.77
45	I acknowledge my mistakes.	3.06	0.92	0.96
46	I actively seek feedback on my performance.	3.12	0.52	0.89
47	I adjust my behavior based on the feedback received.	2.07	1	0.94
48	I accept feedback when it is given to me.	2.03	0.92	0.96
49	When I am unsure, I ask questions and seek guidance.	3.8	0.84	0.94
50	I am curious about acquiring new information, skills, and professional behaviors.	2.52	0.68	0.90
51	I strive to address my knowledge and skill gaps by asking questions of mentors and individual study.	2.97	0.84	0.96
52	I modify my behaviors, skills, and attitudes in response to new learning.	3.06	0.6	0.72
53	I identify my strengths and weaknesses and find strategies to improve my weaknesses.	3.06	0.76	0.81
54	I demonstrate in practice that I prioritize the patient’s interests over my own.	3.45	0.68	0.82
55	I design the environment in a way that can make the patient feel comfortable.	3.12	0.2	0.58
56	I respect cultural differences.	3.52	0.76	0.92
57	I ensure fairness in dealing with different patients regardless of race, religion, beliefs, gender, etc.	3.12	0.92	0.97
58	I provide services to meet people’s needs without regard to their characteristics.	3.59	0.76	0.88
59	I support the patient’s needs when facing system barriers.	3.24	0.2	0.66
60	I use neutral and non-judgmental language.	2.69	0.6	0.76
61	If the patient requires financial resources and social support, I actively work to provide legal assistance for them and their families through social work units or charitable organizations.	4.01	0.28	0.82
62	I go beyond my usual duties to provide care for patients.	3.12	0.28	0.58

*The initial item pool contained 62 items. After removing 13 items during content validity and 3 items during face validity, 3 new items were added based on expert feedback. Additionally, 2 items were split into 4 items to enhance clarity, resulting in a final total of 51 items.

**Table 2:** Questionnaire after CVI & CVR.

Dimensions	Items
**Conscientiousness and responsibility**	I ensure that patient care is followed up at various stages.
	I correctly carry out the assigned educational and clinical tasks.
	I am available to patients.
	I use the healthcare system’s tools and resources appropriately.
	I assist in the training of junior residents and other learners to the best of my ability.
	I accurately document the patient’s condition or the care provided (hospital, clinical, and educational documentation).
	I make decisions about patient treatment based on my professional knowledge, up-to-date scientific evidence, skills, and experiences.
	I carry out and follow up on the care that I have planned for the patient.
	I expedite the medical care of the patient under my supervision.
	I expedite medical consultations in other departments.
	I am available to colleagues.
**Adherence to the laws**	I arrive on time and at the scheduled hour in the department.
	I adhere to the educational policies set by the authorities.
	I submit requests for leave or shift changes in writing in advance.
	In case of absence or delay, I inform the professors, supervisor, colleagues, senior residents, and patients in a timely and appropriate manner.
	I adhere to the dress code regulations.
	I provide the patient with necessary information about the illness in an understandable language and with patience.
	I follow the hospital rules and regulations.
	I have studied the residency training regulations.
	When necessary, I provide the patient’s family with the information about the illness in an understandable language and with patience.
	I act in accordance with the educational and research regulations of the residency program.
**Respect for others**	I treat the patient with respect.
	I provide services to patients with complete respect and without expecting anything in return.
	I respect my colleagues.
	I do not use demeaning language when interacting with others.
	I treat the patient’s companions with respect.
	I maintain appropriate eye contact during the patient examination.
	I listen to the patient’s comments and questions with full attention.
	I maintain my composure in difficult situations.
	I respect the patient’s right to refuse treatment and care.
**Integrity and Honesty**	I treat the patient as a person (not as an object or a case).
	Patient safety is my priority.
	I maintain patient confidentiality.
	I am honest in my interactions with colleagues.
	I am a reliable individual to colleagues and patients.
	Within the framework of my diagnostic and therapeutic responsibilities, I pay attention to the patient’s needs during the examination.
**Professional excellence**	I accept my mistakes.
	I actively seek feedback on my performance.
	I adjust my behavior based on the feedback received.
	When given feedback, I carefully review the aspects and accept it if necessary.
	When I am unsure about the correctness of a subject, I ask questions and seek guidance.
	I actively strive to acquire new information, skills, and professional behaviors.
	I address my knowledge and skill gaps by asking professors.
	I adjust my attitudes, behaviors, and skills in response to new learning.
	I identify my strengths and weaknesses and find strategies to improve my weaknesses.
	I address my knowledge and skill gaps through individual study.
**Altruism**	I demonstrate in practice that I prioritize the patient’s interests over my own.
	I respect cultural differences.
	I uphold fairness in my dealings with patients, regardless of race, religion, belief, gender, etc.
	I provide the necessary services to meet the patients’ needs in the best way, regardless of their personal characteristics.
	I avoid personal judgment in the process of diagnosing and treating patients.

### Face Validity

For qualitative face validity, the opinions of 12 medical residents were assessed, and based on their feedback, minor revisions were made to two items. In the quantitative face validity assessment, after calculating the impact score for all items, three items (14, 21, and 22) scored less than 1.5 and were removed (Table 1).

Overall, following content and face validity evaluations, a total of 16 items were removed, reducing the pool from 62 to 46 items. Subsequently, based on expert recommendations, four new items were added to address conceptual gaps, and two items were split into four separate items to enhance clarity and specificity. This refinement process resulted in a final set of 51 items that were used for construct validity assessment (Table 2).

**Construct Validity**

217 medical residents participated in this phase (Table 3).

**Table 3:** Demographic Characteristics of Medical Residents Participating in Factor Analysis.

Qualitative variables	Groups	Frequencies	Percentage
Gender	Man	106	48.8
	woman	111	51.2
Marital status	single	139	64.1
	married	69	31.8
	Divorced/death of spouse	9	4.1
Number of children	without children	77	35.2
	1-2 children	69	31.5
	More than two children	2	1
Indigenous situation (West Azerbaijan)	yes	125	57.6
	No	92	42.4
Field of study	Internal Medicine	49	22.4
	Pediatric	28	12.8
	Gynecology	24	11
	General Surgery	15	6.8
	Urology	6	2.7
	Pathology	23	10.5
	Orthopedics	16	7.3
	Neurosurgery	11	5
	Neurology	12	5.5
	Cardiology	15	6.8
	Anesthesiology	7	3.2
	Radiology	11	5
Year of residency	First	51	23.5
	Second	61	28.1
	Third	58	26.7
	Fourth	44	20.3
	Fifth	3	1.4
Birthplace	city	207	95.4
	village	10	4.6
Residence	Dorm	31	14.3
	private house	64	29.5
	Parent’s House	32	14.7
	Rental house	84	38.7
	other	6	2.8
Quantitative variables	Minimum	Maximum	Mean ± SD
Age	25	60	33.84 ± 6.92
Number of shifts per month	0	15	8.31 ± 3.34
Monthly income of internship period (dollars)	137	246	223.18 ± 224.12

The results of the factor analysis showed that the KMO sampling adequacy index for the factor analysis model was 0.80, indicating that the sample size was adequate for factor analysis. Bartlett’s test of sphericity was significant (p < 0.001), indicating sufficient correlations among the variables to perform factor analysis. To determine the number of factors underlying the final questionnaire, the scree plot and eigenvalues method were used. The scree plot confirmed the selection of six factors, as the factors beyond the sixth one were approximately at the same level. Thus, six factors are sufficient to explain the factor structure of the Professional Behavior Questionnaire (Fig 1).

**Fig 1:**
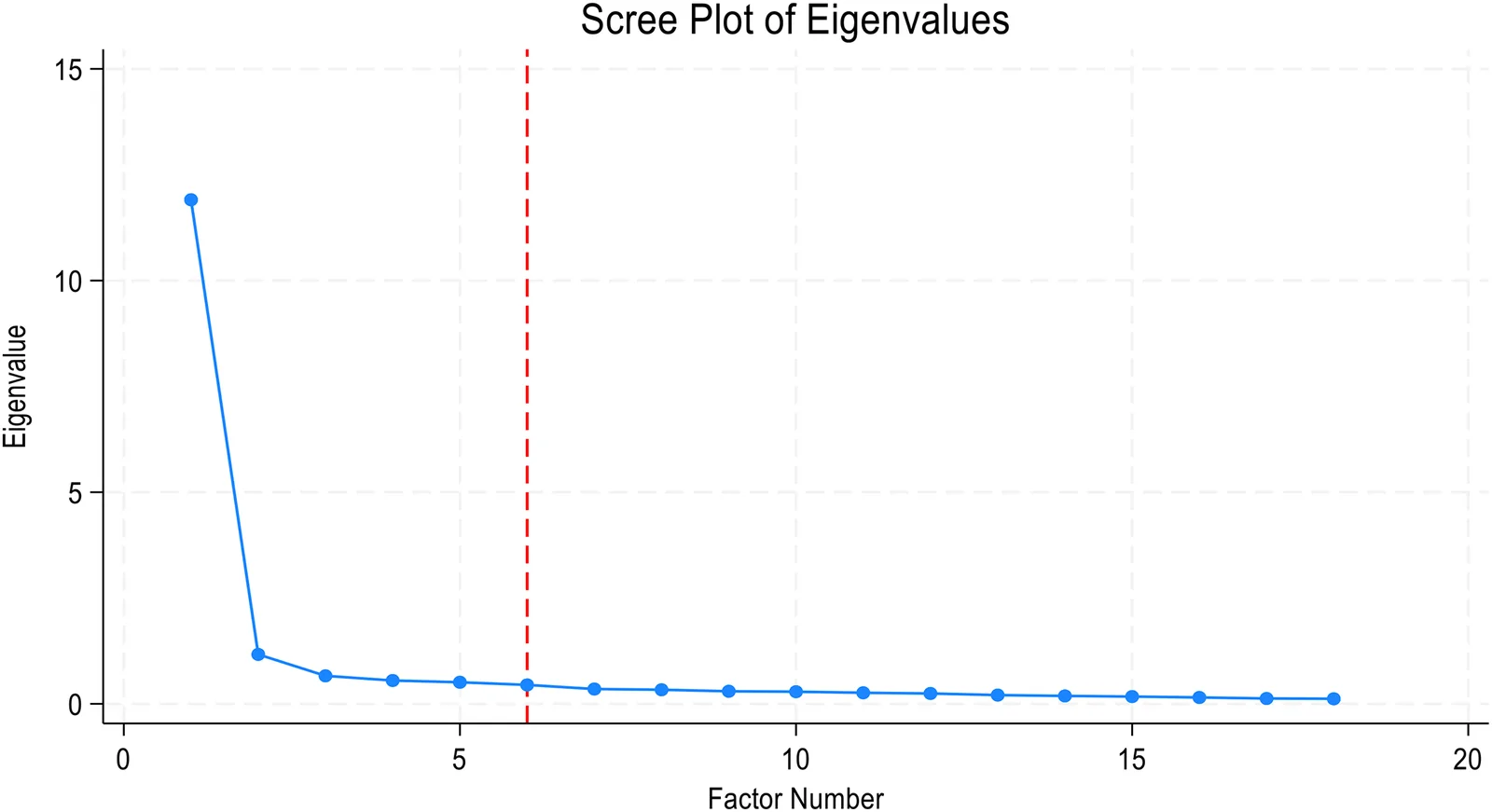
Scree plot showing eigenvalues versus factor numbers. The red vertical dashed line marks the cutoff point at six factors, indicating the elbow where the slope flattens, justifying the retention of the six-factor solution.

The EFA of the 18-item version revealed a six-factor solution accounting for 84.7% of the total variance. The inter-factor correlation matrix (see Table 4) showed moderate-to-strong correlations between factors (r = 0.31–0.64), justifying the use of oblique rotation and reflecting the integrated nature of professional behaviors. The CFA results indicated a good model fit for the six-factor solution: chi^2 = 271.20, text{df} = 120, chi^2/\text{df} = 2.26, Comparative Fit Index (CFI) = 0.961, Tucker–Lewis Index (TLI) = 0.950, Root Mean Square Error of Approximation (RMSEA) = 0.076 (90% CI: 0.064–0.088), and Standardized Root Mean Square Residual (SRMR) = 0.029. All standardized factor loadings were statistically significant (p < 0.001), ranging from 0.812 to 0.924, confirming robust convergent validity for the six latent constructs (Table 5).

**Table 4:** Eigenvalues and Total Explained Variance of the Questionnaire Factors.

Factors	Eigenvalue	Difference	Proportion	Cumulative
1	11.90892	10.73972	0.6616	0.6616
2	1.16920	0.50657	0.0650	0.7266
3	0.66263	0.11013	0.0368	0.7634
4	0.55251	0.04170	0.0307	0.7941
5	0.51080	0.06255	0.0284	0.8224
6	0.44825	0.09762	0.0249	0. 8474

**Table 5:** Standardized Factor Loadings and Measurement Parameters of the 18-Item CFA Model (n = 217).

Latent Construct / Items	Factor Loading (β)	SE	z-value	p-value
Dimension 1: Conscientiousness and Responsibility				
Item 1 (Treatment decisions based on evidence/skills)	0.884	0.024	36.83	< 0.001
Item 2 (Implementation and follow-up of care plan)	0.856	0.027	31.70	< 0.001
Item 3 (Expediting patient care)	0.812	0.031	26.19	< 0.001
Dimension 2: Adherence to the Laws				
Item 4 (Following hospital rules)	0.825	0.029	28.45	< 0.001
Item 5 (Providing patient/family guidance)	0.841	0.028	30.04	< 0.001
Item 6 (Adherence to residency regulations)	0.869	0.025	34.76	< 0.001
Dimension 3: Respect for Others				
Item 7 (Respecting colleagues)	0.892	0.022	40.55	< 0.001
Item 8 (Respecting patients’ companions)	0.865	0.025	34.60	< 0.001
Item 9 (Attentive listening to patients)	0.834	0.028	29.79	< 0.001
Dimension 4: Integrity and Honesty				
Item 10 (Viewing patient as a person)	0.871	0.024	36.29	< 0.001
Item 11 (Honesty with colleagues)	0.853	0.026	32.81	< 0.001
Item 12 (Attention to patient needs during examination)	0.829	0.029	28.59	< 0.001
Dimension 5: Professional Excellence				
Item 13 (Receptivity to feedback)	0.924	0.019	48.63	< 0.001
Item 14 (Active pursuit of knowledge/behaviors)	0.880	0.024	36.67	< 0.001
Item 15 (Self-assessment and strategic improvement)	0.846	0.027	31.33	< 0.001
Dimension 6: Altruism				
Item 16 (Prioritizing patient interests over self)	0.901	0.021	42.90	< 0.001
Item 17 (Respecting cultural differences)	0.862	0.026	33.15	< 0.001
Item 18 (Unconditional care delivery)	0.838	0.028	29.93	< 0.001

*Note: Model Fit Indices: \chi^2 = 271.20, {df} = 120, \chi^2/\text{df} = 2.26, {CFI} = 0.961, {TLI} = 0.950, {RMSEA} = 0.076 [90% CI: 0.064–0.088], {SRMR} = 0.029. All loadings are standardized (\beta) and statistically significant at p < 0.001.*

Following the psychometric analyses, 18 items were retained based on their satisfactory factor loadings, absence of significant cross-loadings, and conceptual coherence within the resulting factor structure. Table 6 presents the reconstructed factor structure for the refined 18-item scale. All factor loadings were within the optimal range (>0.50), and no significant cross-loadings were observed. The final 6-factor structure demonstrated high structural stability. Inter-factor correlations (ranging from r = 0.31 to 0.64) justified the use of oblique rotation over orthogonal methods.

**Table 6:** Pattern Matrix for the Refined 18-Item Medical Professionalism Scale (n = 215).

Dimensions	Items	Factor Loading	Uniqueness
**Conscientiousness and responsibility**	I make decisions about patient treatment based on my professional knowledge, up-to-date scientific evidence, skills, and experiences.	0.874	0.098
	I carry out and follow up on the care plan for the patient.	0.847	0.167
	I expedite the medical care of the patient under my supervision.	0.796	0.117
**Adherence to the Laws**	I follow the hospital rules and regulations.	0.779	0.113
	When necessary, I provide the patient’s family with the information about the illness in an understandable language and with patience.	0.699	0.145
	I act in accordance with the educational and research regulations of the residency program.	0.698	0.115
**Respect for Others**	I respect my colleagues.	0.857	0.152
	I treat the patient’s companions with respect.	0.759	0.169
	I listen to the patient’s comments and questions with full attention.	0.493	0.165
**Integrity and Honesty**	I treat the patient as a person (not an object or a case).	0.433	0.183
	I am honest in my interactions with colleagues.	0.452	0.168
	Within the framework of my diagnostic and therapeutic responsibilities, I pay attention to the patient’s needs during the examination.	0.297*	0.194
**Professional Excellence**	When given feedback, I carefully review the aspects and accept it if necessary.	0.858	0.096
	I actively strive to acquire new information, skills, and professional behaviors.	0.689	0.175
	I identify my strengths and weaknesses and find strategies to improve my weaknesses.	0.581	0.171
**Altruism**	I demonstrate in practice that I prioritize the patient’s interests over my own.	0.845	0.105
	I respect cultural differences.	0.772	0.179
	I provide the necessary services to meet the patient’s needs in the best way, regardless of their characteristics.	0.564	0.228

Note: Extraction Method: Principal Component Analysis. Rotation Method: Promax with Kaiser Normalization. Total variance explained = 84.7%. *Item 36 was retained due to its indispensable conceptual necessity within the Honesty domain and its robust item-rest correlation (r = 0.85).

To ensure optimal readability, Table 6 displays only the representative items with the highest factor loadings for each domain.

### Reliability

To determine the questionnaire’s reliability, both stability and internal consistency were assessed. The test-retest method was employed to evaluate the stability of the instrument. Accordingly, the questionnaire was administered to 20 medical residents twice, with a 10-day interval between administrations. The results of the two assessments were analyzed, yielding an intraclass correlation coefficient (ICC) of 0.83. To assess the internal consistency of the instrument, Cronbach’s alpha coefficient was calculated. In this regard, the questionnaire was completed by 217 participants from the target group. The overall Cronbach’s alpha for all items was found to be 0.94. The Cronbach’s alpha coefficients for the subscales were as follows: Conscientiousness and responsibility 0.91, adherence to the laws 0.89, respect for others 0.93, integrity and honesty 0.93, professional excellence 0.95, and altruism 0.94.

### Scoring and Interpretation

For score interpretation, a criterion-referenced approach was utilized. Mean scores for each domain were calculated on a 5-point Likert scale, where scores between 1.00 and 2.33 indicate low adherence, 2.34 to 3.66 indicate moderate adherence, and 3.67 to 5.00 indicate high adherence to professional standards. Descriptive statistics for the 18-item version are summarized in Table 7.

**Table 7:** Descriptive Statistics and Qualitative Interpretation of Domain Scores (n = 217).

Dimensions	Mean	SD	Min–Max	Interpretation
Conscientiousness and responsibility	4.23	0.68	1.00–5.00	High Adherence
Adherence to the Laws	4.43	0.68	1.00–5.00	High Adherence
Respect for Others	4.56	0.62	1.00–5.00	Very High Adherence
Integrity and Honesty	4.6	0.62	1.00–5.00	Very High Adherence
Professional Excellence	4.42	0.68	1.00–5.00	High Adherence
Altruism	4.5	0.65	1.00–5.00	Very High Adherence
Total Instrument	4.45	0.57	1.00–5.00	High Adherence

*Note: Interpretation categories: 1.00–2.33 (Low Adherence), 2.34–3.66 (Moderate Adherence), 3.67–5.00 (High Adherence).*

As shown in Table 7, the highest mean score was observed in the integrity and honesty domain (Mean = 4.60, SD = 0.62), while responsibility yielded the lowest relative mean (Mean = 4.23, SD = 0.68). However, all domains remained within the ‘High Adherence’ range.

## Discussion

In this study, an instrument was designed and validated to assess the professional behavior of medical residents across six dimensions. The initial draft and item pool of the Professional Behavior Questionnaire, consisting of 62 items, were developed through a comprehensive literature review and an examination of relevant questionnaires assessing professional behavior from the perspectives of patients, colleagues, and faculty members [27,30,38–40]. This diversity of sources contributed to achieving an optimal level of validity and reliability for the questionnaire.

In the content validity assessment, feedback from 25 experts in various fields of health sciences and medicine led to necessary modifications to the questionnaire items. Based on the Content Validity Ratio (CVR), 12 items with a score below 0.37 were removed. Regarding the Content Validity Index (CVI), eight items scored below 0.70, with only one item not overlapping with those removed in the CVR stage. In total, 13 items were excluded. Additionally, based on the CVI results, seven items underwent revisions in wording and phrasing. Adjustments were also made regarding the placement of items, and suggestions for new items were incorporated. For the face validity assessment, based on the feedback from 12 medical residents (the target group), minor revisions were made to two items, and three items with an impact score of less than 1.5 were eliminated. According to experts, the face validity evaluation confirmed the comprehensibility of the questionnaire items for the target group [41]. Consequently, it can be asserted that the designed questionnaire items were clearly worded and easily understood and were well understood, thus confirming face validity.

The construct analysis and scree plot confirmed the selection of six factors: conscientiousness and responsibility, adherence to the laws, respect for others, integrity and honesty, professional excellence, and altruism. These empirically extracted dimensions strongly affirm our initial implicit theoretical proposition, which was guided by the national professionalism guidelines and international literature, suggesting that these six domains adequately capture the complexity of self-reported professional behavior in this cohort. The consistency between our pre-study conceptualization and the post-analysis factor structure significantly reinforces the construct validity of the instrument. The findings align with previous studies and assessment tools used to evaluate professionalism among healthcare professionals [27,42–44]. For instance, Saeedi Tehran et al. (2019), in their study aimed at developing the first medical professionalism guideline in Iran, identified six dimensions: altruism, honor and integrity, responsibility, respect, justice, and excellence ^27^. Similarly, Rakhshan et al. (2020), in their study on developing a professional behavior assessment tool for midwives, highlighted client interaction, personal responsibility, and collaboration with colleagues as key aspects of professional behavior [44]. In another study by Wiggins et al. (2009), which aimed to assess patients’ perceptions of medical residents, professionalism items from the patients’ perspective included addressing patient concerns, showing kindness, understanding patient conditions, and the presence of residents in clinical environments [42]. Some of these professionalism attributes and expectations identified in international studies are also incorporated in the final questionnaire developed in the present study, reflecting the universality of ethical and human values across different cultures and social environments worldwide. On the other hand, specific differences observed in professional behavior expectations may indicate varying societal expectations and distinct characteristics of different communities in their perceptions of physicians’ professionalism.

Beyond comparisons of the identified domains, it is also important to consider how the present instrument differs from established approaches to assessing professionalism. Established professionalism assessment approaches, such as the Professionalism Mini-Evaluation Exercise (P-MEX) and multisource feedback (MSF), provide valuable information about professional behavior from external or observer-based perspectives. The P-MEX, for example, was developed to assess observable professional behaviors in clinical settings and has also been validated in Persian among emergency medicine residents in Iran [45]. Similarly, MSF collects information about observable workplace behaviors from multiple individuals who interact with the learner or physician, providing a broader external perspective on professional performance [46]. In contrast, the present instrument was specifically developed to capture residents’ own perceptions of their professional behavior and the expectations they encounter during residency training. This resident-centered perspective may complement externally based assessment approaches by providing information that may not be directly observable to evaluators, particularly regarding residents’ perceptions of responsibility, adherence to regulations, respect, integrity, excellence, and altruism. Thus, the present instrument should be viewed as complementary to, rather than a replacement for, established observational and multisource assessment methods.

Recent psychometric studies further illustrate the multidimensional nature of professionalism and provide opportunities to compare the present instrument with more recently developed measures across different educational and cultural contexts. For example, Tanrıverdi et al. (2023) validated a 22-item Turkish version of the Professionalism Assessment Scale among medical students, confirming three dimensions of empathy and humanism, professional relationship and development, and responsibility [47]. Similarly, Xin et al. (2024) developed a 23-item self-administered scale for assessing professional behaviors among medical students during clerkships and identified six domains: respect, altruism, communication and collaboration, integrity, duty, and excellence [48]. These findings show substantial conceptual overlap with the six domains identified in the present study, particularly regarding responsibility, respect, integrity, excellence, and altruism. However, differences in the target population and assessment context are important. Unlike these instruments, the present questionnaire was specifically developed from the perspective of medical residents and was grounded in the educational, organizational, and sociocultural context of residency training in Iran. More recently, Li et al. (2025) developed and validated the China Medical Professionalism Inventory through multisite psychometric studies and identified four dimensions encompassing respect, compassion and communication, integrity, excellence, and responsibility [49]. This finding further demonstrates that core dimensions of professionalism may be shared across contexts while their organization and emphasis may vary according to cultural and educational settings. Taken together, these comparisons suggest that the present instrument extends the existing literature by combining broadly recognized dimensions of professionalism with contextually relevant aspects of professional behavior among medical residents in Iran.

The six extracted factors demonstrated strong construct validity, with the model explaining 84.7% of the total variance. This level of explained variance exceeds commonly reported benchmarks in scale development studies and exceeds that reported in several comparable investigations. For example, Goz and Geckil (2010) reported 57.2% of explained variance in the development of a professional behavior scale for nurses [50]. In the present study, exploratory factor analysis of the refined 18-item instrument revealed a clear six-factor structure with all factor loadings exceeding 0.50 and no evidence of cross-loadings, indicating strong structural clarity and stability. These findings were further supported by confirmatory factor analysis, which demonstrated excellent model fit and high standardized factor loadings (0.81–0.92), providing additional evidence of convergent and construct validity. Taken together, the EFA and CFA results indicate that the instrument possesses a well-defined and psychometrically sound factorial structure.

Beyond statistical adequacy, the six-factor structure reflects conceptually meaningful dimensions of professionalism that align with established theoretical frameworks and incorporate culturally grounded nuances specific to Iranian residency training. Although the domains correspond in part to widely recognized elements such as accountability, integrity, respect, and commitment to excellence described by Cruess and Cruess, the ABIM Physician Charter, and Hodges’ multilevel model, the interpretation of these factors extends beyond simple replication [15,28]. Each domain represents the way residents in the Iranian clinical context understand and enact professional behavior within the expectations, hierarchies, and sociocultural values that characterize local medical training environments. For example, the factor adherence to the laws reflects not only ethical responsibility but also the prominent role of hierarchical decision-making and compliance with institutional protocols in Iranian teaching hospitals. The professional excellence domain includes behaviors related to self-improvement and diligence, yet its item content shows how perseverance, duty, and striving despite constraints are culturally valued expressions of professionalism. The respect for others factor illustrates interpersonal professionalism, but the items emphasize culturally salient aspects of communication, deference, and relational harmony. Similarly, responsibility, integrity and honesty, and altruism encompass universal elements of professionalism while simultaneously highlighting context-specific moral expectations, communal responsibility, and culturally embedded norms of modesty and moral conduct. Therefore, although the overarching domains share conceptual foundations with international professionalism models, the resulting factor structure represents a contextually informed operationalization that captures how Iranian residents experience, interpret, and enact professional behavior. This indicates that the instrument does not merely reproduce existing frameworks but provides novel, locally grounded insights into the behavioral dimensions of professionalism in residency training.

This study demonstrated strong reliability indices for the developed instrument. The overall Cronbach’s alpha of 0.94 indicates exceptionally high internal consistency, and the test–retest intraclass correlation coefficient (ICC = 0.83) reflects good temporal stability. These findings align with widely accepted psychometric standards, which indicate that alpha values above 0.70 and ICC values above 0.75 denote acceptable reliability [41,51]. Consistent with prior research on professionalism assessment tools such as instruments developed or validated by lots of studies [30,50,52,53], the present instrument similarly demonstrates robust reliability. Nevertheless, the exceptionally high Cronbach’s alpha requires careful interpretation, as very high coefficients may reflect item redundancy. To address this concern, confirmatory factor analysis (CFA) was conducted, which led to a reduction in the number of items and improved the conceptual coherence of the instrument. This refinement helps minimize potential redundancy and reduces respondent burden, particularly among residents with demanding clinical workloads, while maintaining strong psychometric properties.

Taken together, the factor structure identified in this study demonstrates that professionalism in Iranian residency training is shaped not only by universal ethical principles but also by culturally and institutionally embedded norms. Although the six domains parallel widely recognized professionalism frameworks, their behavioral expressions reflect context-specific characteristics such as hierarchical clinical structures, strong expectations for adherence to the laws, and cultural values emphasizing respect, modesty, and communal responsibility. These findings align with prior evidence showing that the manifestation of professionalism varies across sociocultural environments [29]. Incorporating the resident perspective further enriches the conceptual interpretation, as residents experience ethical tensions, workload pressures, and hidden curriculum influences that may not be readily captured through patient or faculty evaluations. Nevertheless, because the instrument was developed and tested within a single institution, the extent to which these culturally shaped expressions generalize to other educational or cultural settings remains uncertain. Future research should therefore examine the instrument across multiple institutions and diverse cultural contexts to evaluate the stability of the factor structure and identify components that may require contextual refinement. Such efforts will strengthen the cross-cultural applicability and broader utility of the instrument.

### Limitations and Strengths

This study has several limitations that should be considered when interpreting the findings. First, data were collected from a single medical university in Iran using a census approach that included all eligible residents at Urmia University of Medical Sciences. Although this approach provided comprehensive coverage of the eligible resident population at the institution and the sample size was adequate for exploratory factor analysis, the single-institution setting limits the generalizability of the findings and the psychometric properties of the instrument to other regions, institutions, or cultural environments. Future studies should validate the instrument using larger, multicenter samples from different regions and medical universities in Iran to examine the stability of its factor structure and other measurement properties across diverse residency programs. Cross-cultural validation in other countries and cultural contexts would also be valuable to determine whether the instrument’s factor structure and psychometric properties are transferable or require contextual adaptation. Second, although content and face validity were established using both qualitative and quantitative methods, including evaluation by 25 experts and feedback from 12 residents, these processes inherently involve subjective judgment. Broader expert panels from additional specialties and cultural backgrounds may strengthen the comprehensiveness of item assessment. Third, test–retest reliability was examined using a relatively small sample of 20 residents over a 10-day interval. Although the ICC value of 0.83 suggests good stability, the limited sample size restricts the precision of this estimate. Replication with larger and more heterogeneous samples is recommended. Fourth, the study relied exclusively on positively worded self-report items, which increases the risk of social desirability bias, an especially important concern in professionalism research, where respondents may feel compelled to portray themselves favorably. This bias may have inflated scores or reduced variability, particularly within hierarchical residency environments. Future studies should incorporate strategies to mitigate this bias, such as using reverse-scored items, employing social desirability scales, or supplementing self-report data with multisource feedback, direct observation, or patient ratings. Finally, the cross-sectional nature of the study limits the ability to examine changes in professionalism over time or establish temporal relationships between professionalism scores and subsequent outcomes. Longitudinal research and predictive validity studies are needed to assess how professionalism scores relate to subsequent clinical performance, behavioral outcomes, or residency milestones.

Despite these limitations, this study has several strengths that enhance its contribution to medical education research in Iran. First, to our knowledge, there is currently no validated instrument for assessing residents’ and physicians’ professionalism from their own perspective within the Iranian context. By developing and psychometrically evaluating such a tool, this study addresses an important gap in the national literature and provides a foundation for future research and educational interventions. Second, the instrument was developed through a rigorous mixed-methods process, including item generation based on qualitative analysis, expert review for content and face validity, exploratory factor analysis, and assessment of predictive and test–retest reliability. These steps reinforce the methodological robustness of the tool. Third, the inclusion of residents from multiple specialties enhances the relevance and applicability of the instrument across various clinical disciplines. Finally, the resulting six-factor model offers a clear, coherent, and practical structure that can inform both assessment practices and curriculum development related to professionalism in medical residency programs.

## Conclusion

The 18-item questionnaire developed in the present study is a valid and reliable tool for assessing the professional behavior of medical residents and general medical students in clinical settings. Given the fundamental importance and significant impact of medical residents’ professional behavior on public health and the provision of high-quality healthcare services, it is recommended that educators, administrators, and healthcare system stakeholders use this questionnaire to evaluate the professional behavior of medical residents. Based on the findings, necessary interventions and actions should be designed and implemented to strengthen and address any identified weaknesses in their professional conduct.

## Supporting information

S1 Table Questionnaire for assessing Professional Behavior. (DOCX)

S1 Dataset The study dataset. (CSV)

## Abbreviations

ICC: Intraclass Correlation Coefficient of Reliability
CBSM: Community-based Social Marketing
CVR: Content Validity Ratio
CVI: Content Validity Index
EFA: Exploratory Factor Analysis
CFA: Confirmatory factor analysis
CFI: Comparative Fit Index
TLI: Tucker-Lewis Index
RMSEA: Root Mean Square Error of. Approximation
SRMR: Standardized Root Mean Square Residual
KMO: Kaiser–Meyer–Olkin

## References

[1] Pereira I, Cunningham AM, Moreau K, Sherbino J, Jalali A. Thou shalt not tweet unprofessionally: An appreciative inquiry into the professional use of social media. Postgrad Med J. 2015;91(1080):561–4. doi: 10.1136/postgradmedj-2015-133353 26294333 PMC4621375

[2] Yavari N, Asghari F, Shahvari Z, Nedjat S, Larijani B. Obstacles of professional behavior among medical trainees: A qualitative study from Iran (2018). J Educ Health Promot. 2019;8:193. doi: 10.4103/jehp.jehp_272_19 31807585 PMC6852278

[3] Ellaway RH, Chou CL, Kalet AL. Situating Remediation: Accommodating Success and Failure in Medical Education Systems. Acad Med. 2018;93(3):391–8. doi: 10.1097/ACM.0000000000001855 28767496

[4] Rosenstein AH. Physician disruptive behaviors: Five year progress report. World J Clin Cases. 2015;3(11):930–4. doi: 10.12998/wjcc.v3.i11.930 26601095 PMC4644894

[5] Cruess RL, Cruess SR. Teaching medicine as a profession in the service of healing. Acad Med. 1997;72(11):941–52. doi: 10.1097/00001888-199711000-00009 9387815

[6] Papadakis MA, Hodgson CS, Teherani A, Kohatsu ND. Unprofessional behavior in medical school is associated with subsequent disciplinary action by a state medical board. Acad Med. 2004;79(3):244–9. doi: 10.1097/00001888-200403000-00011 14985199

[7] Shumway JM, Harden RM, Association for Medical Education in Europe. AMEE Guide No. 25: The assessment of learning outcomes for the competent and reflective physician. Med Teach. 2003;25(6):569–84. doi: 10.1080/0142159032000151907 15369904

[8] Cruess SR, Johnston S, Cruess RL. “ Profession”: A working definition for medical educators. Teaching learn med. 2004;16(1):74–6. 10.1207/s15328015tlm1601_15 14987179

[9] Hilton SR, Slotnick HB. Proto-professionalism: How professionalisation occurs across the continuum of medical education. Med Educ. 2005;39(1):58–65. doi: 10.1111/j.1365-2929.2004.02033.x 15612901

[10] Jha V, Bekker HL, Duffy SRG, Roberts TE. Perceptions of professionalism in medicine: A qualitative study. Med Educ. 2006;40(10):1027–36. doi: 10.1111/j.1365-2929.2006.02567.x 16987195

[11] Swick HM. Toward a normative definition of medical professionalism. Acad Med. 2000;75(6):612–6. doi: 10.1097/00001888-200006000-00010 10875505

[12] Van De Camp K, Vernooij-Dassen MJFJ, Grol RPTM, Bottema BJAM. How to conceptualize professionalism: A qualitative study. Med Teach. 2004;26(8):696–702. doi: 10.1080/01421590400019518 15763872

[13] Wagner P, Hendrich J, Moseley G, Hudson V. Defining medical professionalism: A qualitative study. Med Educ. 2007;41(3):288–94. doi: 10.1111/j.1365-2929.2006.02695.x 17316214

[14] Hafferty FW. Definitions of professionalism: A search for meaning and identity. Clin Orthop Relat Res. 2006;449:193–204. doi: 10.1097/01.blo.0000229273.20829.d0 16770288

[15] Hodges BD, Ginsburg S, Cruess R, et al. Assessment of professionalism: recommendations from the Ottawa 2010 Conference. Medical teacher. 2011;33(5):354–63. 10.3109/0142159X.2011.577300 21517683

[16] Goldie J. Assessment of professionalism: A consolidation of current thinking. Med Teach. 2013;35(2):e952-6. doi: 10.3109/0142159X.2012.714888 22938675

[17] Asghari F, Shahvari Z, Ebadi A, Alipour F, Samadi S, Bahreini M, et al. Developing and validating an instrument to measure: the medical professionalism climate in clinical settings. J Med Ethics Hist Med. 2021;14:11. doi: 10.18502/jmehm.v14i11.7666 35035799 PMC8696557

[18] Kelley KA, Stanke LD, Rabi SM, Kuba SE, Janke KK. Cross-validation of an instrument for measuring professionalism behaviors. Am J Pharm Educ. 2011;75(9):179. doi: 10.5688/ajpe759179 22171107 PMC3230340

[19] Li H, Ding N, Zhang Y, Liu Y, Wen D. Assessing medical professionalism: A systematic review of instruments and their measurement properties. PLoS One. 2017;12(5):e0177321. doi: 10.1371/journal.pone.0177321 28498838 PMC5428933

[20] Cruess RL, Cruess SR. Teaching professionalism: general principles. Med Teach. 2006;28(3):205–8. doi: 10.1080/01421590600643653 16753716

[21] Cruess SR, Cruess RL. Professionalism and medicine’s social contract with society. Virtual Mentor. 2004;6(4):virtualmentor.2004.6.4.msoc1-0404. doi: 10.1001/virtualmentor.2004.6.4.msoc1-0404 23260480

[22] Blank L, Kimball H, McDonald W, Merino J, ABIM Foundation, ACP Foundation, et al. Medical professionalism in the new millennium: a physician charter 15 months later. Ann Intern Med. 2003;138(10):839–41. doi: 10.7326/0003-4819-138-10-200305200-00012 12755556

[23] Norcini J, Anderson MB, Bollela V, Burch V, Costa MJ, Duvivier R, et al. 2018 consensus framework for good assessment. Med Teach. 2018;40(11):1102–9. doi: 10.1080/0142159X.2018.1500016 30299187

[24] Terwee CB, Mokkink LB, Knol DL, Ostelo RWJG, Bouter LM, de Vet HCW. Rating the methodological quality in systematic reviews of studies on measurement properties: a scoring system for the COSMIN checklist. Qual Life Res. 2012;21(4):651–7. doi: 10.1007/s11136-011-9960-1 21732199 PMC3323819

[25] van Mook WNKA, van Luijk SJ, O’Sullivan H, Wass V, Schuwirth LW, van der Vleuten CPM. General considerations regarding assessment of professional behaviour. Eur J Intern Med. 2009;20(4):e90-5. doi: 10.1016/j.ejim.2008.11.011 19524166

[26] Wilkinson TJ, Wade WB, Knock LD. A blueprint to assess professionalism: results of a systematic review. Acad Med. 2009;84(5):551–8. doi: 10.1097/ACM.0b013e31819fbaa2 19704185

[27] Saeedi Tehrani S, Nayeri F, Parsapoor A, et al. Development of the first guideline for professional conduct in medical practice in Iran. Archives of Iranian medicine. 2017;20(1):0–0. 28112525

[28] Cruess R, McIlroy JH, Cruess S, Ginsburg S, Steinert Y. The Professionalism Mini-evaluation Exercise: a preliminary investigation. Acad Med. 2006;81(10 Suppl):S74-8. doi: 10.1097/00001888-200610001-00019 17001141

[29] Chandratilake M, McAleer S, Gibson J. Cultural similarities and differences in medical professionalism: A multi-region study. Med Educ. 2012;46(3):257–66. doi: 10.1111/j.1365-2923.2011.04153.x 22324525

[30] Hajibabaee F, Moghimi Darzi SK, Khatami F, Ashrafizadeh H, Shojaei A. Evaluation of psychometric properties of the tool for identifying “Patients’ Views on the Observance of Professional Behavior by Specialized Physicians”. J Ardabil Univ Med Sci. 2021;21(3):329–39. doi: 10.52547/jarums.21.3.329

[31] Sattarzadeh Badkoubeh, Roya, Asghari F, Shahvari Z. Designing and Validating the Tool for Assessing the Professional Behavior of Clinical Faculty Members; A Tool in Stakeholder’s View. Iranian J Med Educ. 2019;19(0):199–212.

[32] Vasheghani Farahani A, Maleki M, Golpira R, et al. Perspectives of doctors, nurses and patients on physicians’ observance of professional ethics. Iranian J Med Ethics History Med. 2015;8(1):59–66.

[33] Lacasse Y, Godbout C, Sériès F. Health-related quality of life in obstructive sleep apnoea. Eur Respir J. 2002;19(3):499–503. doi: 10.1183/09031936.02.00216902 11936529

[34] Hyrkäs K, Appelqvist-Schmidlechner K, Oksa L. Validating an instrument for clinical supervision using an expert panel. Int J Nurs Stud. 2003;40(6):619–25. doi: 10.1016/s0020-7489(03)00036-1 12834927

[35] Gravetter FJ, Forzano L-AB, Rakow T. Research methods for the behavioral sciences. Wadsworth Cengage Learning Belmont, CA; 2009.

[36] Fawcett J, Garity J. Evaluating research for evidence-based nursing practice: FA Davis; 2008.

[37] Ezzy D. Qualitative analysis: Routledge; 2013.

[38] Razavipoor M, Moradi S, Amuei F, Shafizad M, Mahmoodi E, Mohamadalizadeh P. The Evaluation of The Professionalism of Clinical Teachers from The Viewpoint of Residents of Educational Departments of Imam Khomeini Hospital of Mazandaran University of Medical Science. J Med Educ Dev. 2021;13(40):85–94. doi: 10.52547/edcj.13.40.85

[39] Badkoubeh S, Asghari F. Designing and Validating the Tool for Assessing the Professional Behavior of Clinical Faculty Members; A Tool in Stakeholder’s View. Iranian J Medical Educ. 2019;19:199–212.

[40] Hajibabaee F, Moghimi DSK, Khatami F, Ashrafizadeh H, Shojaei AA. Assessing the Behavior and Professional Commitment of Specialist Physicians from Patients’, Perspectives. J Med Spirit Cultivat. 2022;31(2):86–97.

[41] Polit DF, Beck CT. Nursing research: Generating and assessing evidence for nursing practice. Lippincott Williams & Wilkins; 2017.

[42] Wiggins MN, Coker K, Hicks EK. Patient perceptions of professionalism: implications for residency education. Med Educ. 2009;43(1):28–33. doi: 10.1111/j.1365-2923.2008.03176.x 19148978

[43] Abadel FT, Hattab AS. Patients’ assessment of professionalism and communication skills of medical graduates. BMC Med Educ. 2014;14:28. doi: 10.1186/1472-6920-14-28 24517316 PMC3923249

[44] Rakhshan M, Rabiei L, Afshar L, Ayazi Z, Ahmady S. Psychometric evaluation of a professional behavior questionnaire of midwives working in the delivery wards of hospitals under the supervision of shahrekord university of medical sciences, Shahrekord, Iran, in 2019. J Clinical Nurs Midwifery. 2020;9(3):742–52.

[45] Amirhajlou L, Bidari A, Alipour F, Yaseri M, Vaziri S, Rezai M, et al. Validity, reliability and acceptability of Professionalism Mini-Evaluation Exercise (P-MEX) for emergency medicine residency training. J Med Ethics Hist Med. 2019;12:12. doi: 10.18502/jmehm.v12i12.1641 32328225 PMC7166245

[46] Lockyer J, Sargeant J. Multisource feedback: an overview of its use and application as a formative assessment. Can Med Educ J. 2022;13(4):30–5. doi: 10.36834/cmej.73775 36091727 PMC9441111

[47] Tanrıverdi EÇ, Nas MA, Kaşali K, Layık ME, Abd El-Aty AM. Validity and reliability of the Professionalism Assessment Scale in Turkish medical students. PLoS One. 2023;18(1):e0281000. doi: 10.1371/journal.pone.0281000 36701346 PMC9879428

[48] Xin C, Song X, Wang S, Cui X, Ding N, Wen D. Assessing professional behaviors: a self-administered scale for medical students during clerkships. BMC Med Educ. 2024;24(1):692. doi: 10.1186/s12909-024-05676-9 38926701 PMC11200818

[49] Li H, Hirsh DA, Song X, Krupat E, Yang X, Ho M-J, et al. East meets west: A multisite validity study of the china medical professionalism inventory. Perspect Med Educ. 2025;14(1):603–18. doi: 10.5334/pme.1682 41019845 PMC12466328

[50] Goz F, Geckil E. Nursing students professional behaviors scale (NSPBS) validity and reliability. Pak J Med Sci. 2010;26(4):938–41.

[51] Koo TK, Li MY. A guideline of selecting and reporting intraclass correlation coefficients for reliability research. J Chiropr Med. 2016;15(2):155–63. doi: 10.1016/j.jcm.2016.02.012 27330520 PMC4913118

[52] Bajwa NM, Nendaz MR, Posfay-Barbe KM, Yudkowsky R, Park YS. A meaningful and actionable professionalism assessment: Validity evidence for the professionalism mini-evaluation exercise (P-MEX) across 8 years. Acad Med. 2021;96(11S):S151–7. doi: 10.1097/ACM.0000000000004286 34348372

[53] Taşçı Aİ, Akdeniz E, Gülpınar MA, Danacıoğlu YO, Sarı EE, Yaşar L, et al. Adaptation of the professionalism mini-evaluation exercise instrument into Turkish: a validity and reliability study. BMC Med Educ. 2023;23(1):698. doi: 10.1186/s12909-023-04675-6 37752458 PMC10523623

