# Supplementary material for: Development and psychometric properties of a questionnaire to evaluate the professional behavior of medical residents
Source: PLoS One. 2026 Sep 28;21(9):e0358787. doi: 10.1371/journal.pone.0358787 (PMC13618930; doi:10.1371/journal.pone.0358787)
Supplement: Supplementary file 1 [file pone.0358787.s001.docx]

**S1 Table. Questionnaire for assessing Professional Behavior**

| **Row** | **Dimensions and Items** | **Answer** | | | | | |
| --- | --- | --- | --- | --- | --- | --- | --- |
|  |  | Strongly disagree | Disagree | | No Idea | Agree | Strongly agree |
| **Conscientiousness and responsibility** | |  |  |  | |  |  |
| 1 | I make decisions about patient treatment based on my professional knowledge, up-to-date scientific evidence, skills, and experiences. |  |  |  | |  |  |
| 2 | I carry out and follow up on the care plan for the patient. |  |  |  | |  |  |
| 3 | I expedite the medical care of the patient under my supervision. |  |  |  | |  |  |
| **Adherence to the Laws** | |  |  |  | |  |  |
| 4 | I follow the hospital rules and regulations. |  |  |  | |  |  |
| 5 | When necessary, I provide the patient's family with the information about the illness in an understandable language and with patience. |  |  |  | |  |  |
| 6 | I act in accordance with the educational and research regulations of the residency program. |  |  |  | |  |  |
| **Respect for Others** | |  |  |  | |  |  |
| 7 | I respect my colleagues. |  |  |  | |  |  |
| 8 | I treat the patient's companions with respect. |  |  |  | |  |  |
| 9 | I listen to the patient's comments and questions with full attention. |  |  |  | |  |  |
| **Integrity and Honesty** | |  |  |  | |  |  |
| 10 | I treat the patient as a person (not an object or a case). |  |  |  | |  |  |
| 11 | I am honest in my interactions with colleagues. |  |  |  | |  |  |
| 12 | Within the framework of my diagnostic and therapeutic responsibilities, I pay attention to the patient’s needs during the examination. |  |  |  | |  |  |
| **Professional Excellence** | |  |  |  | |  |  |
| 13 | When given feedback, I carefully review the aspects and accept it if necessary. |  |  |  | |  |  |
| 14 | I actively strive to acquire new information, skills, and professional behaviors. |  |  |  | |  |  |
| 15 | I identify my strengths and weaknesses and find strategies to improve my weaknesses. |  |  |  | |  |  |
| **Altruism** | |  |  |  | |  |  |
| 16 | I demonstrate in practice that I prioritize the patient's interests over my own. |  |  |  | |  |  |
| 17 | I respect cultural differences. |  |  |  | |  |  |
| 18 | I provide the necessary services to meet the patient's needs in the best way, regardless of their characteristics. |  |  |  | |  |  |
